# TCR Triggering Induces the Formation of Lck–RACK1–Actinin-1 Multiprotein Network Affecting Lck Redistribution

**DOI:** 10.3389/fimmu.2016.00449

**Published:** 2016-10-27

**Authors:** Ondřej Ballek, Jan Valečka, Martina Dobešová, Adéla Broučková, Jasper Manning, Pavel Řehulka, Jiří Stulík, Dominik Filipp

**Affiliations:** ^1^Laboratory of Immunobiology, Institute of Molecular Genetics AS CR, Prague, Czech Republic; ^2^Faculty of Military Health Sciences, Institute of Molecular Pathology, Hradec Králové, Czech Republic

**Keywords:** TCR triggering, RACK1, Lck, membrane redistribution, lipid rafts, α-actinin, cytoskeleton

## Abstract

The initiation of T-cell signaling is critically dependent on the function of the member of Src family tyrosine kinases, Lck. Upon T-cell antigen receptor (TCR) triggering, Lck kinase activity induces the nucleation of signal-transducing hubs that regulate the formation of complex signaling network and cytoskeletal rearrangement. In addition, the delivery of Lck function requires rapid and targeted membrane redistribution, but the mechanism underpinning this process is largely unknown. To gain insight into this process, we considered previously described proteins that could assist in this process *via* their capacity to interact with kinases and regulate their intracellular translocations. An adaptor protein, receptor for activated C kinase 1 (RACK1), was chosen as a viable option, and its capacity to bind Lck and aid the process of activation-induced redistribution of Lck was assessed. Our microscopic observation showed that T-cell activation induces a rapid, concomitant, and transient co-redistribution of Lck and RACK1 into the forming immunological synapse. Consistent with this observation, the formation of transient RACK1–Lck complexes were detectable in primary CD4^+^ T-cells with their maximum levels peaking 10 s after TCR–CD4 co-aggregation. Moreover, RACK1 preferentially binds to a pool of kinase active pY394^Lck^, which co-purifies with high molecular weight cellular fractions. The formation of RACK1–Lck complexes depends on functional SH2 and SH3 domains of Lck and includes several other signaling and cytoskeletal elements that transiently bind the complex. Notably, the F-actin-crosslinking protein, α-actinin-1, binds to RACK1 only in the presence of kinase active Lck suggesting that the formation of RACK1–pY394^Lck^–α-actinin-1 complex serves as a signal module coupling actin cytoskeleton bundling with productive TCR/CD4 triggering. In addition, the treatment of CD4^+^ T-cells with nocodazole, which disrupts the microtubular network, also blocked the formation of RACK1–Lck complexes. Importantly, activation-induced Lck redistribution was diminished in primary CD4^+^ T-cells by an adenoviral-mediated knockdown of RACK1. These results demonstrate that in T cells, RACK1, as an essential component of the multiprotein complex which upon TCR engagement, links the binding of kinase active Lck to elements of the cytoskeletal network and affects the subcellular redistribution of Lck.

## Introduction

Signaling through the T-cell antigen receptor (TCR) has the potential to trigger a broad range of cellular responses (1). During TCR triggering, two Src family tyrosine kinases (SFKs) – Lck and Fyn – provide critical enzymatic and structural functions that predicate the generation of the most proximal signals emanating from the TCR (2). In CD4^+^ resting T-cells, Lck is targeted to the inner leaflet of the plasma membrane *via* its NH_2_-terminal myristate/palmitate motif. A considerable portion of this membrane-associated Lck has been shown to be non-covalently attached to the TCR co-receptor, CD4 (3). Lck kinase activity is positively and negatively regulated by the phosphorylation of two tyrosine residues, Y394 and Y505, respectively, the former being associated with fully active Lck (4). Upon TCR binding to a cognate peptide which is recognized in the context of MHCII, CD4 interacts with the non-variable region of the same MHCII and juxtaposes its bound kinase active Lck within the vicinity of immunoreceptor tyrosine-based activation motifs (ITAMs) of the CD3 chains of TCR. Lck then phosphorylates ITAMs that serve as docking sites for activated tyrosine kinase ZAP-70, which in turn proceeds to phosphorylate the adaptor protein LAT at multiple sites. This leads to the recruitment of downstream signaling elements such as phospholipase C-γ1 and adaptor proteins Grb2 and GADS which trigger complex signaling cascades, Ca^2+^ flux, cytoskeletal reorganization, and integrin activation (5, 6).

There is a general consensus that a T-cell membrane structural network provides the necessary milieu for coordination and integration of processes that regulate the onset of T-cell signaling. Several types of membrane heterogeneities that concentrate specific and distinct sets of signaling molecules have been proposed. These account for, but are not limited to, lipid rafts (LRs), nanoclusters, protein islands, pickets and fences, transient confinement zones, microclusters, immunological synapse (IS), and supramolecular activation cluster (SMAC) (7). LRs, which represent a sizable fraction of the plasma membrane, are in terms of their composition, structure, and function among the most studied (8, 9). Due to their enrichment in cholesterol and sphingolipids, LRs exist in a liquid-ordered phase, hence are largely resistant to solubilization by mild non-ionic detergents, and can be isolated as detergent-resistant membrane (DRM) fractions. While DRMs are not equated with native LRs, their content and properties allow the examination of changes in membrane raft content induced by TCR signaling (10–13).

The compartmentalization of membrane-residing signaling proteins into LRs provides the basis for their physical segregation and transient clustering (14). Two distinct types of DRM fractions have been documented in resting T-cells: light and heavy DRMs, which are enriched for non-overlapping subsets of signaling molecules (15). Importantly, TCR activation-induced LR redistribution of Lck and several other signaling molecules which are involved in the initiation of signaling cascades, such as CD3ζ, LAT, and CD45, have been documented (14).

While TCR triggering is enzymatically initiated by Lck-mediated tyrosine phosphorylation of CD3 ITAMs, Lck does not remain in a stationary position. There are several lines of evidence that demonstrate that the delivery of Lck function is accompanied by its rapid and targeted membrane redistribution. Notably, we previously reported that LR plays an essential role in temporal and spatial coordination and activation-dependent redistribution of Lck and Fyn kinases (16, 17). A proposed Lck-dependent Fyn activation model posits that antibody-mediated TCR–CD4 co-aggregation-induced Lck activation outside LR results in Lck translocation to light LR where the activation of LR-resident Fyn ensues. Similarly, the “Lck standby model” which does not specifically account for the existence of LR, proposes that upon TCR triggering, the constitutively kinase active fraction of membrane-bound Lck is targeted to areas where it colocalizes with ITAMs of TCR/CD3 complex (18). Studies by Rossy et al. also demonstrated the impact of TCR stimulation on Lck distribution, which was dependent on active conformation of the kinase (19). Furthermore, it has been reported that the early redistribution of Lck to the forming IS with its maximum kinase activity occurs between 2 and 5 min after initiation of T-cell–APC conjugation (20). While these data collectively established Lck as a mobile signaling element that is indispensable for proximal T-cell signaling (21), the underlying process regulating its redistribution is currently unknown. The main aim of the study was to gain insight into the molecular mechanism and its functional elements that regulate the early recruitment of Lck to LR and the formation of the IS.

To consider proteins that could act in aiding the process of activation-induced redistribution of Lck, we searched for previously described molecules with the following attributes: (i) regulation of intracellular translocation of kinases, (ii) ability to interact with SFKs and modulate their kinase activity, (iii) capacity to associate with elements of the cytoskeletal network, and (iv) capacity to interact with multiple partners. Among several candidates, receptor for activated C kinase 1 (RACK1) turned out to be a viable option. RACK1 is a 36-kDa scaffolding protein, which is evolutionary highly conserved in a wide range of eukaryotes including members of the plant, fungi, and yeast kingdoms (22). It is expressed in all mammalian cells, and its deficiency is embryonically lethal (23). It contains seven WD40 repeats (24) with a propeller structure, which provides RACK1 with multiple binding sites allowing interaction with a large number of functionally and structurally distinct proteins such as G proteins, kinases, phosphatases, and IP3 receptors (25). This interactivity predisposes RACK1 to be involved in a broad range of cellular processes, from signal transduction, transcription, translation, viral infection, cell migration, development, and epigenetics to cancer (25). Its interactions with protein kinase C (PKC) (26), membrane-bound receptors such as integrin β (27, 28), NMDA receptor (29), FAK, PDE, and SFKs such as Src and Fyn (30–32) have been studied in detail. Importantly, it has been shown that RACK1 can positively or negatively modulate the kinase activity of SFK Src, Fyn, and Lck, with the resulting effect being cell context specific (27–33). However, the specific role of RACK1 in proximal T-cell signaling has not been investigated so far.

An important and yet enigmatic function of RACK1 is the coordinated translocation and redistribution of its activated binding partners to a distinct subcellular compartment (25). While mechanisms regulating these dynamic processes are still obscure, the multiple protein-binding capacity of RACK1’s seven WD40 domains and its association with beta-spectrin and/or plectin which mediate interactions with major cytoskeletal elements, actin, intermediate filaments, and possibly microtubules most likely contribute to these processes (34, 35). Interestingly, RACK1 is also a constituent of the ribosomal assembly where it recruits signaling components that enable the regulation of translation (36, 37).

Here, we report microscopic, biochemical, and genetic data characterizing RACK1 as an integral part of signaling transduction network capable of transiently co-binding the activated Lck and elements of cytoskeleton, thus revealing its potential in aiding Lck redistribution by integrating TCR/CD4–Lck signals with filament networks.

## Materials and Methods

### Mice

Three mice strains, 6–8 weeks old, were used: wild-type (WT) C57BL/6, OTII transgenic mice with transgenic (tg) TCRα/TCRβ receptor specific for OVA peptide (38), and double tg DO11.10 TCR/tg CARΔ (TgCAR) (Taconic), which express human coxsackie/adenovirus receptor lacking a cytosolic domain (39, 40). All the three strains were housed in a specific pathogen-free animal facility at the Institute of Molecular Genetics (Prague, Czech Republic).

### Antibodies and Reagents

For western blotting, mouse anti-RACK1, rabbit anti-pY394^Lck^ (Santa Cruz), anti-pY505Lck (Cell signaling), mouse anti-Lck (3A5), and phosphotyrosine-specific platinum 4G10 mAb (Millipore) were used. Mouse anti-α-actinin-1 (BM-75.2), rabbit anti-RACK1 C-end, anti-GAPDH, and cholera toxin–horseradish peroxidase (HRP) conjugate were purchased from Sigma-Aldrich, anti-α-tubulin from GeneTex, and anti-GADS and anti-LASP from Millipore. Mouse anti-rabbit and goat anti-mouse HRP light chain-specific antibodies (Jackson IRLab) were used for protein detection after immunoprecipitation (IP). For IP purposes, antibodies were coupled to Protein A or G magnetic beads (Millipore) or the bead-conjugated anti-RACK1 from Santa Cruz were used. Biotinylated anti-mouse CD4 (GK1.5) and biotinylated anti-mouse TCRβ (H57) mAbs were purchased from eBiosciences (USA). For immunofluorescence, CellTrace™ Far Red DDAO-SE and Alexa Fluor 488, 555, or 647 goat anti-mouse IgG or goat anti-rabbit IgG (H + L) were used. Actin filaments were visualized by Alexa Fluor 488 Phalloidin purchased from Life Technologies. Streptavidin, Brij58 (polyoxyethylene-20-cetyl-ether), and nocodazole were purchased from Sigma-Aldrich. Latrunculin B, lauryl maltoside (*n*-dodecyl-β-d-maltoside, LM), and Src-family kinase inhibitor PP2 were obtained from Calbiochem (Merck). Ultrapure grade paraformaldehyde (PFA) was obtained from Polysciences. OVA_323–339_ peptide from GenScript Corporation, and staphylococcal enterotoxin E (SEE) was purchased from Toxin Technology.

### Cell Lines, Cell Preparation, and Culture

The NIH3T3 cell line and Lck infectants cell lines were maintained in Iscove’s modified Dulbecco’s medium (IMDM), WT, and JCAM1.6 The Jurkat T-cell line and RAJI B-cell line were cultured in RPMI 1640 (Life Technologies). Both media were supplemented with 10% inactivated fetal calf serum (FCS) and 100 U of penicillin/10 μg streptomycin antibiotics (Sigma) per 1 ml of media. Bone marrow-derived dendritic cells (BMDCs) from OTII transgenic mice were isolated from mice femur and tibia cavities. The cells were cultured for 6 days in RPMI medium supplemented with GM-CSF-containing supernatant, which was produced by the LUTZ cell line (final concentration was adjusted to 30 ng/ml). After 3 days of cultivation, one half of the media was replenished, and on day 6, the cells were harvested and used for further experiments.

### cDNA Vectors

Lck constructs, described previously (21), were inserted into the murine stem cell virus (MSCV)-based internal ribosome entry site (IRES)-enhanced green fluorescent protein virus MigR1 (41), permitting the concurrent expression of a given gene and EGFP. EGFP–RACK1 and mCitrine–RACK1 was a gift from Dr. Vomastek, and CFP–Lck (clone W25) was a gift from Dr. Stockinger (42). Generation of retrovirus packaging cell lines and retrovirus stock as well as the infection of NIH3T3 or Jurkat T-cells was performed as outlined elsewhere (43, 44). Successfully infected cells were sorted to the comparable protein expression level of the desired gene.

### Isolation and Activation of Primary T-Cells

The procedure was performed as previously described (14). Briefly, primary lymph node CD4^+^ T-cells (~95% purity) were isolated from mice using MACS CD4^+^ T-cell isolation kit (AutoMACS, Miltenyi Biotec). CD4^+^ T-cells were precoated with biotinylated anti-TCR (1 μg/ml, clone H57) and anti-CD4 (0.3 μg/ml, clone GK1.5) antibodies in 500 μl of PBS + 3% FCS for 30 min at 4°C, washed, and resuspended in 20 μl of PBS + 1% FCS per tube or were indicated in 20 μl of PBS + 1% FCS + 20 μM PP2. Cells were pre-warmed in 37°C, and co-aggregation-mediated activation was achieved with the addition of streptavidin to a final concentration of 50 μg/ml. Cells were vortexed and incubated at 37°C for the indicated period of time. The activation was stopped with the addition of either ice-cold lysis buffer with indicated detergent and inhibitors (see below) or immediately boiled in Laemmli sample buffer.

### Cell Lysis and Immunoprecipitation

Cell lysis of CD4^+^ T-cells was performed in TKM (50 mM Tris, pH 7.4, 25 mM KCl, 5 mM MgCl_2_, 1 mM EDTA, 0.5% Brij58) or TNE (50 mM Tris, pH 8, 12.5 mM EDTA, 1% NP-40) lysis buffer supplemented with protease and phosphatase inhibitor cocktails (Roche). Lysates were incubated on ice for 30 min, spun down at 800 × *g* for 2 min (removal of nuclei), and used for further applications. For RACK1 IPs, the lysates were incubated with antibody-precoated RACK1 beads overnight at 4°C on a rotational wheel. Beads were then intensively washed (six to eight times), boiled in Laemmli sample buffer for 10 min, and immunoprecipitated proteins resolved by SDS-PAGE.

### Western Blotting and Quantification

Proteins resolved on polyacrylamide gels were transferred onto a PVDF membrane (Millipore) and blocked for 1 h in 5% non-fat milk (for IP) or in 1% BSA in TBS-T at room temperature. Blots were then incubated for 1 h with primary antibodies diluted in blocking buffer, washed followed by a 1-h incubation with secondary antibodies conjugated to HRP, and developed by incubation with ECL substrate (Thermo Scientific). Where indicated, densitometry quantification was performed by AIDA image analyzer software from raw image data obtained from a GS-800 Biorad densitometer scanner.

### Immunofluorescence Microscopy

For immunofluorescence, the staining protocol was adopted from the website www.cellsignal.com with some modifications. Briefly, CD4^+^ T-cells were seeded on poly-l-lysine-coated coverslips and allowed to adhere for 15 min at 37°C, 5% CO_2_ followed by cell fixation in 4% PFA for 15 min. The following procedure was applied to all cell types. After PFA fixation, the cells were permeabilized with ice-cold methanol for 10 min in −20°C (methanol step had to be omitted when phalloidin was used to stain actin cytoskeleton). The cells were blocked for 1 h with PBS containing 0.3% Triton X-100 (PBT) with the addition of 2.5% FCS and 2.5% BSA and incubated with primary and secondary antibodies for 1 h consecutively. Where indicated, cells were stained with DAPI for 10 min. Coverslips were mounted using 4% *n*-propyl gallate in glycerol. Samples were analyzed by sp5 confocal microscopy (Leica) or N-SIM super-resolution microscopy (Nikon). Image reconstruction was performed by Huygens Professional (SVI) or NIS elements (Nikon) software. Image post-editing and image analysis including computing of Pearson’s colocalization coefficient (PCC) were done with Fiji imaging software and its plug-in JACoB.

### Preparation of APC–CD4^+^ T-Cell Conjugates

The preparation of conjugates for microscopic analysis of IS formation *in vitro* was performed as previously described (45). Briefly, BMDCs were prepared in parallel as described above. Cells were then pulsed with OVA_323–339_ peptide for 2 h and TCR-transgenic CD4^+^ primary T-cells isolated from OTII mice were admixed with APCs at 3:1 ratio. The formation of APC–T-cell conjugates was achieved by short centrifugation. Conjugates were incubated in serum-free RPMI medium at 37°C. The cells were fixed with 4% PFA for 15 min, stained with anti-RACK1 and anti-Lck, and used for the microscopy analysis.

### Formation of RAJI–Jurkat-Cell Conjugates, Live Cell Imaging

The WT or Lck-deficient Jurkat T-cell line JCAM1.6 was retrovirally transfected with either an EGFP–RACK1 or CFP–Lck construct, respectively. In addition, the mCitrine–RACK1 construct was electroporated (BTX ECM 830, 300 V, 10 ms, 4 mm cuvette) into CFP–Lck stable infectants. Jurkat T-cells that were positive for both CFP and mCitrine were sorted (BD, Influx cell sorter) and rested for 1 day in 37°C/CO_2_. The formation of RAJI–Jurkat conjugates using live cell imaging was performed as previously described (46). Briefly, RAJI cells, serving as APCs, were labeled with DDAO-SE (Life Technologies), then loaded with 1 μg/ml SEE, and transferred into cover glass chamber (Ibidi). CFP–Lck/mCitrine–RACK1 Jurkat T-cells were subsequently added at a 1:1 ratio. Cells were observed in a 37°C/CO_2_ climate chamber using a DeltaVision Core/Olympus IX71 microscope under CFP/YFP/mCherry filter cubes. Images were acquired every 15 s. Image post-editing and time-lapse movies were done with Fiji imaging software.

### Quantification of Fluorescence in Microscopic Images

To analyze the concentricity and apposition of RACK1 and Lck (or GADS), the distance from the centroid of the cell to the cell edge was measured using a fluorescent intensity profile (see details in Figure S1 in Data Sheet 1 in the Supplementary Material). All microscopic quantitative analyses were performed by ImageJ program. Statistical analyses were performed with GraphPad Prism 5 using a paired two-tailed *t*-test.

Quantitation of the recruitment of RACK1 to the IS was calculated as described previously (47). Briefly, using the selection brush tool in ImageJ program, the T cell area adjacent to the synapse region, outside synapse region and the background area outside of the cell, was demarcated. The relative recruitment index (RRI) was calculated as the [mean fluorescence intensity (MFI) at synapse region minus the background]/[MFI of the outside synapse region minus background]. Quantitative measurements of MFI were performed with the program ImageJ. Statistical analysis was performed with GraphPad Prism 5 using a paired two-tailed *t*-test.

### Gel Filtration

This size-exclusion chromatography procedure is based on a previously described protocol (48) with slight modifications (14). Briefly, a 5-ml pipette tip plugged with a small piece of glass wool and filled with Sepharose 4B beads (Sigma-Aldrich) was used as the column. Cells were lysed in TKM-Brij58 lysis buffer for 30 min and spun down for 2 min at 800 × *g*. Supernatant at a total volume of 1/10 of the stationary bead volume was loaded onto the top of the column and eluted with cell lysis buffer. All steps were performed at 4°C. In this setting, fraction #4 contains complexes of >10^7^ Da; most of the pentameric IgM and IgG standards eluted in fractions #7 and #9, respectively (48).

### Isolation of Detergent-Resistant Membranes

The isolation of DRMs was performed as described previously (14, 16). For the assessment of protein distribution in fractions obtained by gel filtration, pooled fractions were mixed 1:1 with 80% sucrose and subjected to the same protocol. Light DRMs, corresponding to classical LRs, are enriched in top fractions (#1–3), while the bottom fractions (#8–10) concentrate heavy DRMs together with soluble proteins.

### Cytosketal Inhibitors

For assessing the involvement of cytoskeletal components in the redistribution of Lck after TCR/CD4 aggregation, CD4^+^ T-cells were resuspended in PBS + 3% FCS and treated with either 2 μg/ml of latrunculin B or 10 μM of nocodazole for 30 min in 37°C and 5% CO_2_. Subsequent precoating of the cells and TCR/CD4 co-aggregation were also performed in the presence of these inhibitors on ice. Before activation, small aliquots of latrunculin B- and nocodazole-treated samples, as well as untreated controls sample, were pre-warmed and fixed at 37°C with PFA for 15 min, stained as described above, and analyzed by microscopy.

### Adenoviral Vectors and Transduction of TgCAR T-Cells

Adenoviral vectors and virus particles containing shRNA hairpins, as well as control empty vector/viruses, were prepared using the Knockout RNAi system and the Adeno-X Expression system 1 (Clontech) and used according to the manufacturer’s protocol. Target sequences of shRNA for RACK1 are as follows: RAO#2-gcaagatcattgtagatgaat, RAO#4-ctcccacttcgttagtgat, and RAO#5-ggatgagagtcattcagaatg. Transduction of T-cells was performed as described elsewhere (49). Briefly, isolated naïve CD4^+^ T-cells were resuspended in DMEM medium containing 2% FCS and desired MOI of adenoviral particles and incubated at 37°C/CO_2_ for 1 h. Then, the cell/virus mixture was transferred to a culture dish and incubated for the indicated time at 37°C/CO_2_ in complete RPMI media supplemented with 2 ng/ml of rmIL-7 (PeproTech). Then, live T-cells were sorted, rested for 4 h, and used for further experiments.

### Mass Spectrometry

The stained protein bands were processed according to the standard protocol generally used for mass spectrometry (MS) protein identification (50) with minor modifications. The gel slices containing the proteins of interest were washed, proteins reduced with dithiothreitol, alkylated with iodoacetamide, and digested with trypsin. The extracted peptides were separated using a home-made microgradient device (51) with C18 reversed phase capillary column (i.d. 200 μm, length 70 mm) for LC MALDI-TOF/TOF MS and MS/MS analysis using 4800 Proteomics Analyzer (Applied Biosystems, Framingham, MA, USA) with α-cyano-4-hydroxycinnamic acid as MALDI matrix. Protein database identification was carried out with Protein Pilot 2.0 software using the SwissProt part of the UniProt database server.

## Results

### Localization of Lck and RACK1 in CD4^+^ Primary T-Cells

**Figure 1:**
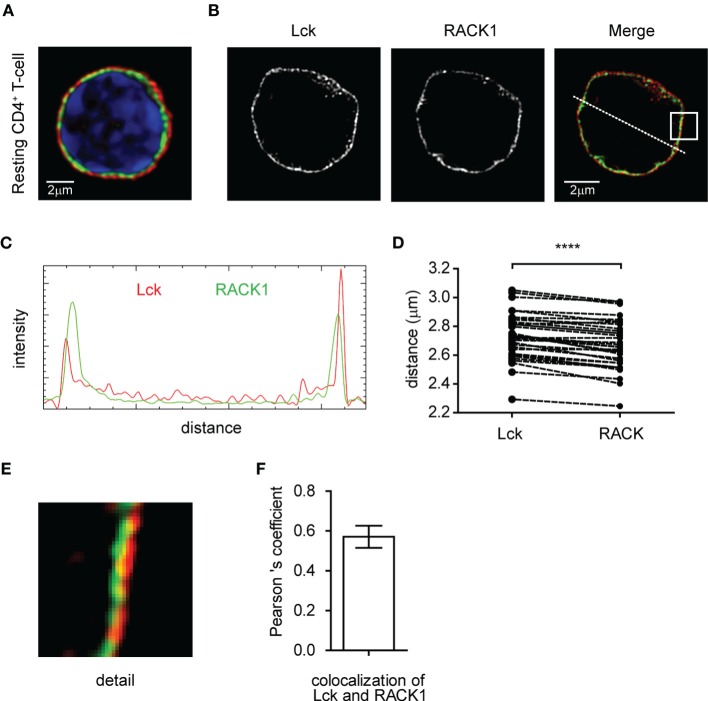
**Subcellular distribution of Lck and RACK1 in primary CD4^+^ T-cell**. Fixed CD4^+^ T-cells were stained for RACK1 (green), Lck (red), and nuclei (blue) and visualized by confocal microscopy **(A)** or super-resolution N-SIM microscopy **(B)**. **(C)** Fluorescence intensity profile plot of Lck (red) and RACK1 (green) along the dotted line shown in the merged image of figure **(B)**. **(D)** Statistical analysis of the concentric juxtaposition of Lck and RACK1, which shows a larger distance of Lck from the cell centroid to its periphery (*n* = 30), *p* ≤ 0.0001. **(E)** Magnification of the rectangle inset from the Merge image presented in **(B)** showing a subconcentrical juxtaposition of RACK1 to membrane-bound Lck. **(F)** The bar graph represents the statistical analysis of Lck and RACK1 colocalization using Pearson’s colocalization coefficient (*n* = 20 cells). Error bars denote SD.

First, we determined the expression of RACK1 in mouse primary lymph node CD4^+^ T-cells. RT-PCR analysis performed on total mRNA isolated from FACS-sorted cells (99.3% purity) demonstrated detectable levels in non-activated CD4^+^ T-cells (data not shown). Confocal microscopy confirmed the presence of RACK1 on the protein level and showed that Lck is localized almost exclusively to the plasma membrane. We observed that RACK1 is positioned just beneath Lck, in a constrained cytoplasmic niche between the plasma membrane and nucleus, the latter fulfilling the vast majority of intracellular space (Figure 1A). To assess RACK1 subcellular distribution more accurately, we performed super-resolution microscopy. It confirmed that in resting CD4^+^ T-cells, Lck and RACK1 are juxtaposed concentrically (Figures 1B–D) and exhibit a mild overlap (Figure 1E) in accordance with the PCC >0.6 (Figure 1F).

### RACK1 and Lck Co-Redistribute to Forming Immunological Synapse

**Figure 2:**
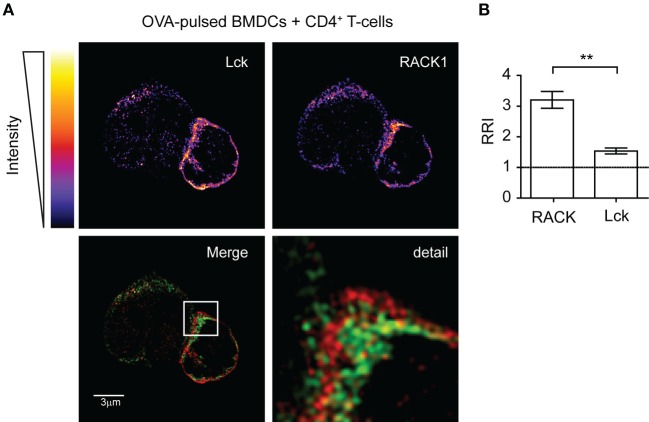
**Lck and RACK1 in primary CD4^+^ T-cell co-redistribute into forming immunological synapse**. **(A)** OVA-pulsed bone marrow-derived dendritic cells (BMDCs) were mixed with CD4^+^ T-cells from OTII transgenic mice. After 2–3 min, T-cell–APC conjugates were seeded on cover slips, fixed and probed with anti-Lck (red) and anti-RACK1 (green), and visualized by super-resolution N-SIM microscopy. The upper two panels show the fluorescence intensities of individual Lck or RACK1 signals using pseudocolor digital scaling. The panel titled “Detail” shows the magnification of the rectangle inset seen in the Merge image. An image of one representative cell conjugate is presented. Consistent with a previous report (52), the low expression of Lck was also detected in BMDCs. **(B)** Statistical analysis of the relative recruitment index (RRI) showed that while both RACK1 and Lck are enriched at the site of the forming IS, RRI for the former is significantly higher than that for Lck (*n* = 20), *p* < 0.01.

Next, we assessed the cellular co-distribution of RACK1 and Lck in activated primary lymph node CD4^+^ T-cells during early phases of IS formation. Microscopic examination of cell conjugates of OVA peptide-pulsed BMDCs with transgenic T-cells specific for OVA peptide showed that Lck and RACK1 concomitantly translocated to and enriched in IS at early phases of its formation (2–5 min) (Figures 2A,B). Lck, the accumulation of which in IS has been previously demonstrated (20, 45), was used as an internal control. Interestingly, we observed that RRI for RACK1 is even higher than that for Lck, strongly suggesting the physiological importance of RACK1 enrichment in the forming IS (Figure 2B).

**Figure 3:**
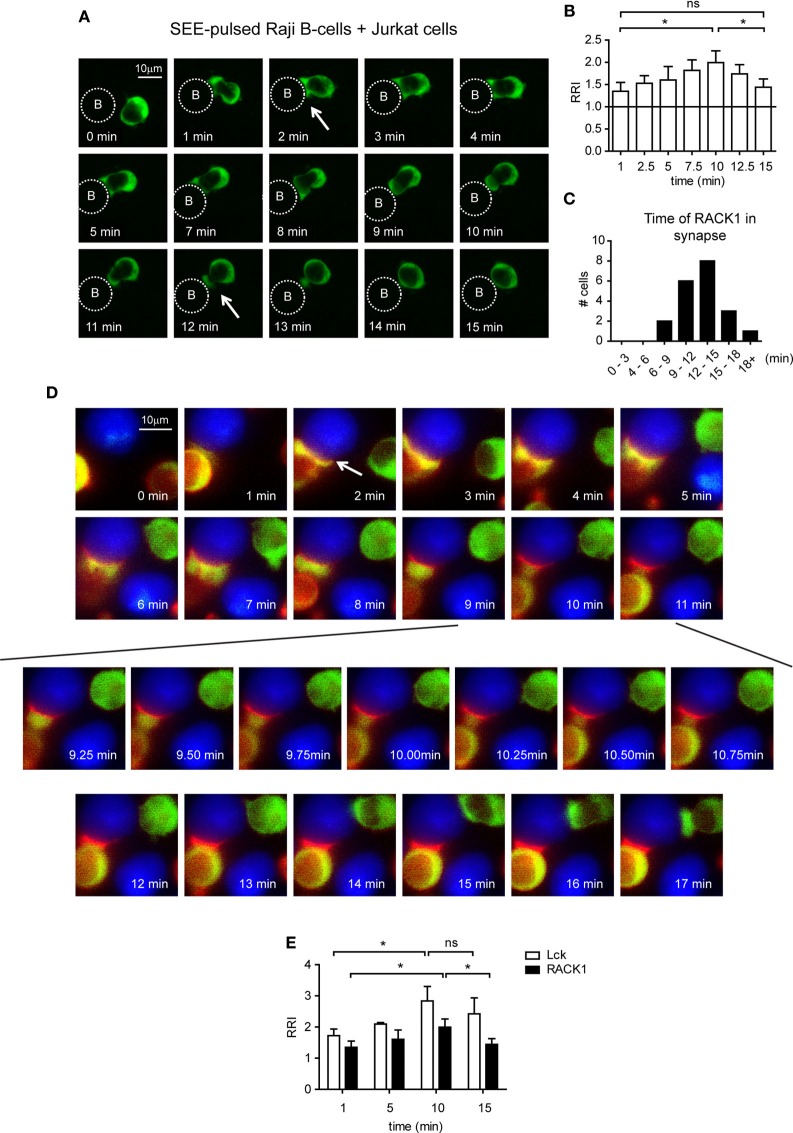
**The kinetics of Lck and RACK1 co-redistribution into forming immunological synapses (IS) in Jurkat T-cells**. **(A)** Jurkat T-cells expressing RACK1–EGFP (green) were mixed with SEE-pulsed RAJI B-cells (denoted by the dotted circle), and the redistribution of RACK1 was observed by live cell imaging microscopy. Sequential time-lapse fluorescence microphotographs from one representative movie (Video S1 in Supplementary Material) are shown. **(B)** Statistical analysis of the kinetics of the relative recruitment index (RRI) of RACK1 to the forming IS measured after cell contact initiation (*n* = 20). **(C)** The bar graph shows the time distribution of RACK1 residency in IS; the start and end points of RACK1 residency are marked by two white arrows shown in **(A)**; *n* = 20 cells. **(D)** Lck-deficient JCAM1.6 Jurkat T-cells co-expressing Lck–CFP (red) and RACK1–mCitrine (green) constructs were mixed with SEE-pulsed RAJI B-cells (blue). Their redistribution during the formation of IS was observed by live cell imaging microscopy. Sequential time-lapse fluorescence microphotographs from one representative movie (Video S2 in Supplementary Material) are shown. An arrow points to the forming IS. **(E)** Statistical analysis of the kinetics of the relative recruitment index (RRI) of RACK1 and Lck to the forming IS measured after cell contact initiation (*n* = 20).

To analyze the co-redistribution of RACK1 and Lck during early phases of IS formation in more detail, we performed experiments that would visualize the kinetics of this process. Toward this end, Jurkat T-cells, which are able to form cell conjugates with RAJI cells were infected with a RACK1–EGFP retroviral construct, and the kinetics of RACK1–EGFP translocation into IS was examined in a time-dependent manner using live fluorescent microscopy (Figure 3A; Video S1 in Supplementary Material). RACK1–EGFP protein was found moderately enriched in the forming IS (Figure 3B) with the total time of its transient residency from 6 min to more than 18 min (Figure 3C).

To visualize the Lck and RACK1 co-redistribution event, we co-transfected Jurkat T-cells with two constructs: RACK1–mCitrine and Lck–CFP. mCitrine and CFP double-positive cells were FACS-sorted, and time-lapse images were recorded (Video S2 in Supplementary Material). As presented in Figure 3D, shortly after conjugate formation (1–2 min, see the arrow in the 2 min time frame), RACK1 and Lck co-redistributed to the forming IS where they both persisted for several minutes. Between 6 and 8 min, RACK1–mCitrine slowly moved distally from IS. At the 9-min mark until 11 min (Figure 3D, bottom two rows of images), suddenly and rapidly RACK1–mCitrine re-translocated back to the bulk volume of cytoplasmic space of the T-cell, leaving Lck behind in the IS where it remained for at least 7–8 min (Figure 3E). It is of note that the Jurkat T-cell which appears in the upper right hand corner of the 2-min image of the time-lapsed video and image and in which the amount of RACK1–mCitrine markedly exceeded that of Lck–CFP (red color is below the visible range) displays similar kinetics of RACK1 translocation to the IS and reverse re-translocation back to the cytoplasm as described above (Figure 3D; Video S2 in Supplementary Material).

Taken together, these data suggest that T-cell activation induces a rapid, cooperative, and IS-directed movement of Lck and RACK1. This co-redistribution pattern provided the first evidence that RACK1 could participate in the mechanism guiding the redistribution of membrane Lck upon TCR engagement.

### RACK1–Lck Complex Formation in the Primary CD4^+^ T-Cells

**Figure 4:**
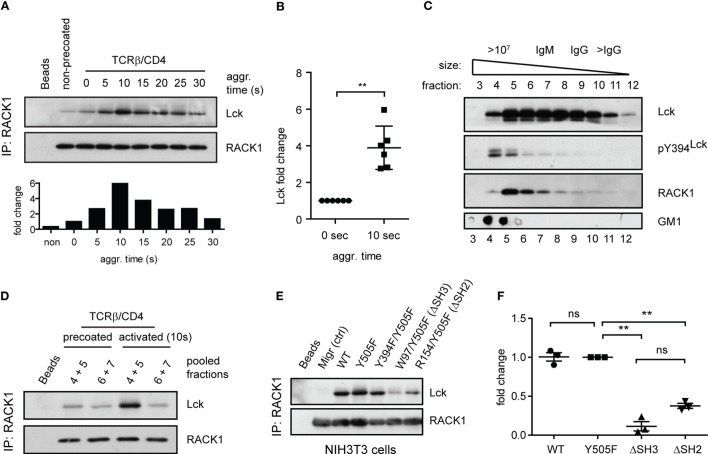
**Antibody-mediated engagement of TCR/CD4 receptors induces RACK1–Lck complex formation in primary CD4^+^ T-cells**. **(A)** CD4^+^ T-cells precoated or non-precoated with biotinylated anti-TCR and anti-CD4 mAbs (TCRβ/CD4) were co-aggregated, or not (0 s), with streptavidin for the indicated time. RACK1 immunoprecipitates were blotted against Lck and RACK1. The bar graph at bottom shows the relative amount of co-immunoprecipitated Lck after normalization to RACK1. **(B)** The graph plot presents the relative fold increase of co-immunoprecipitated Lck (10 s after activation) from six independent experiments. The statistical analysis presented as mean ± SD was performed using the Student’s two-tailed *t*-test, **p < 0.01. **(C)** RACK1–Lck interaction is localized into HMW fractions. CD4^+^ T-cells fractions (#3–12) obtained from a Sephadex column were probed with anti-Lck, anti-pY394^Lck^, anti-RACK1, and cholera toxin B subunit–HRP, which detects the surrogate marker of light DRMs, GM1. The fraction elution profile of the molecular weight size marker is shown on the top. **(D)** Fractions #4–5 and #6–7 from non-activated (precoated) or activated (TCRβ/CD4, 10 s) CD4^+^ T-cells were pooled, immunoprecipitated with anti-RACK1, and blotted with anti-Lck and RACK1 antibody. **(E)** RACK1–Lck interaction depends on functional SH2 and SH3 Lck domains. Endogenously expressed RACK1 was immunoprecipitated from NIH3T3 Lck infectants and blotted with anti-Lck and anti-RACK1 antibodies. **(F)** Statistical analysis of **(E)** represents the relative fold-change of RACK1 co-immunoprecipitated Lck variants from three independent experiments. The statistical analysis presented as mean ± SD was performed using the Student’s two-tailed *t*-test, **p < 0.01, n.s., not significant. Blots shown in **(C–E)** are representatives of at least three independent experiments.

**Figure 5:**
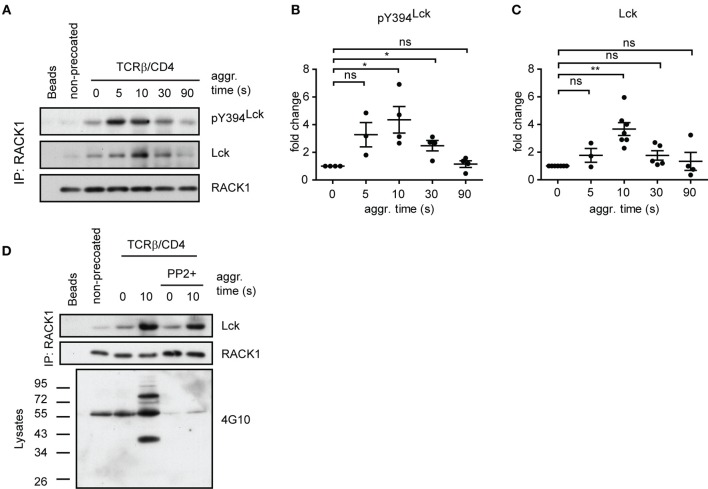
**RACK1–Lck complexes can form irrespective of Lck kinase activity, but those from activated T-cells contain a sizeable fraction of pY394^Lck^**. **(A)** CD4^+^ T-cells precoated or non-precoated with biotinylated anti-TCR and anti-CD4 mAbs (TCRβ/CD4) were co-aggregated, or not (0 s), with streptavidin for the indicated period of time. RACK1 immunoprecipitates were blotted against pY394^Lck^, total Lck, and RACK1. **(B,C)** Statistical analysis of pY394Lck and total Lck blots from **(A)**, respectively, represent the normalized to total RACK1 from at least three independent experiments. The statistical analysis presented as mean ± SD was performed using the Student’s two-tailed *t*-test, *p < 0.05, **p < 0.01, n.s., not significant. **(D)** CD4^+^ T-cells were pretreated, or untreated, with SFK inhibitor (PP2+), TCRβ/CD4 co-aggregated, and lysed in TNE lysis buffer. RACK1 was immunoprecipitated and aliquots blotted against total Lck and RACK1; total cell lysates were blotted with anti-pY 4G10 antibody. Blots shown in **(A,D)** are representative of at least three independent experiments.

To determine if RACK1 is involved in proximal T-cell signaling *via* its interaction with Lck and if it is constitutive or activation inducible, we utilized the model of antibody-mediated T-cell activation enabling to study the kinetics of interaction between two interacting proteins during the first seconds after TCR/CD4 co-aggregation (14, 16) (Figure 4A). On average, a threefold to sixfold enrichment of Lck in complexes with RACK1 reached its maximum level 10 s after TCR–CD4 engagement (Figure 4B). Then, in the following 30–90 s, these levels diminished to those observed in non-activated cells (Figures 4A and 5A,C). Consistent with the colocalization analysis (Figures 1C–F) only low, background levels of RACK1–Lck complexes were detected in the non-precoated control sample (Figures 4A and 5A, “non-precoated” lane).

### Subcellular Distribution of RACK1–Lck Complexes

We recently demonstrated that a small preactivated pool of pY394^Lck^, which plays an important role in TCR triggering in resting primary CD4^+^ T-cells, associates with an LM-sensitive type of membrane microdomains called heavy DRMs (14). This high molecular weight (HMW) fraction can be obtained from Brij58 detergent-solubilized cells by gel filtration (53, 54). Interestingly, it is the heavy DRM-associated pool of Lck which, upon TCR/CD4 engagement, not only significantly increases its kinase activity but also exclusively translocates to distinct types of microdomains called light DRMs (14, 55). These considerations led us to assess the subcellular distribution of RACK1 in CD4^+^ T-cells. As illustrated in Figure 4C, RACK1, similar to the pool of pY394^Lck^, is enriched in the complexes that are associated with high HMW fractions #4–6. Next, we assessed the physical association of Lck and RACK1 in the fractions that were prepared from resting and activated T-cells. As shown in Figure 4D, the immunopreciptation of RACK1 from pooled HMW fractions #4 + 5 and #6 + 7 confirmed the activation-induced formation of RACK1–Lck complexes that were detected only in fractions #4 + 5 that were prepared from activated T-cells. These results suggest that in resting T-cells, the pool of preactivated pY394^Lck^ and RACK1 are in physical proximity by co-distributing to heavy DRMs, which spatially restricts their transient interaction upon TCR activation. These data thus support the prediction that upon TCR/CD4 triggering, RACK1 can bind a spatially restricted pool of kinase active Lck and functions as a transportation vehicle that assists the redistribution of Lck to light DRMs, as documented previously (14, 16).

### Structure–Function Analysis of RACK1–Lck Interaction

Next, we sought to determine which domain or tyrosine residue, which regulates Lck kinase activity, mediates its interaction with RACK1. Toward this end, we prepared a NIH3T3 fibroblast cell line expressing wild type Lck (WT), constitutively active Lck (Y505F) and Y505F Lck backbone with additionally inactivated either SH3 (W97K) or SH2 (R154K) domain (Figures S2A–C in Data Sheet 1 in the Supplementary Material). Endogenous RACK1 was immunoprecipitated with anti-RACK1 antibody, and its aliquots were blotted for Lck and RACK1. We found that in NIH3T3 cells, the WT, Y505F, and even kinase compromised Y505F/Y394F Lck interacted comparably with endogenous RACK1. However, this complex formation was nearly abolished in the variant expressing non-functional SH3 domain and severely compromised in the SH2 mutant of Y505F Lck (Figures 4E,F). This suggests that the presence of both functional modular SH3 and SH2 domains of Lck is a prerequisite for RACK1–Lck complex formation.

### RACK1 Complexes with Lck Regardless of Lck Activation Status

**Figure 6:**
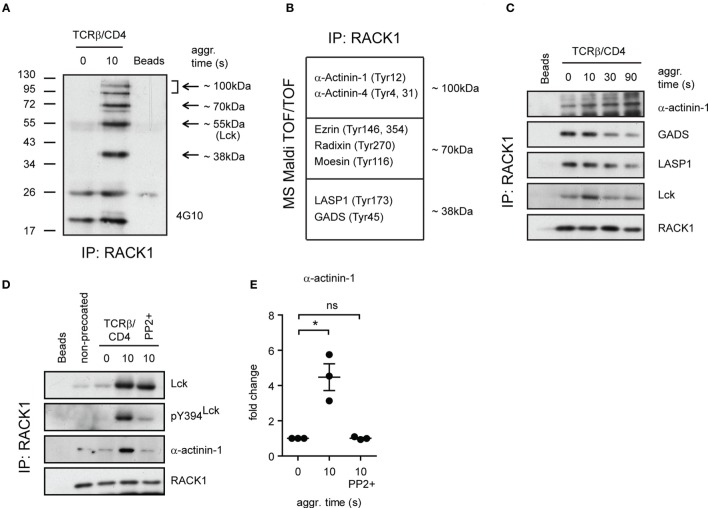
**Identification of additional components of RACK1–Lck complexes**. **(A)** RACK1 immunoprecipitates from non-activated (0 s) and activated (10 s) CD4^+^ T-cell samples, and beads alone, were probed with anti-pY antibody (4G10). The arrows point to areas that show readily detectable phosphoproteins co-immunoprecipitating with RACK1. In the coomassie blue-stained gel, the areas depicted by arrows from all three samples were extracted (except for the 55-kDa area) and subjected to MALDI TOF/TOF MS analysis. A selected list of size-related and identified proteins with their potential pY sites are shown in **(B)**. The bead sample was negative for these proteins. **(C)** The kinetics of complex formation between RACK1 and indicated proteins before (0) and at the indicated time points after TCRβ/CD4 co-aggregation. RACK1 immunoprecipitates were probed with anti-α-actinin-1, anti-GADS, anti-LASP, anti-Lck, and anti-RACK1. **(D)** CD4^+^ T-cells were treated (PP2+) or not with PP2 inhibitor, activated, and lysed in TNE lysis buffer. RACK1 was immunoprecipitated, and samples were blotted against Lck, pY394^Lck^, α-actinin-1, and RACK1. **(E)** Statistical analysis of **(D)**, actinin-1 panel, represents the relative fold change of RACK1 co-immunoprecipitated actinin-1 normalized to total RACK1. The statistical analysis presented as mean ± SD was performed using the Student’s two-tailed *t*-test, *p < 0.05, n.s., not significant. Blots shown in **(C,D)** are representative of three independent experiments.

The above results revealed that RACK1–Lck complex formation, at least in fibroblast cells, proceeds irrespective of the activation status of Lck (Figure 4E, Y505F versus Y394F/Y505F Lck). We next assessed if such a mode of interaction would also occur in primary CD4^+^ T-cells. As shown in Figure 5A, the kinetics of TCR/CD4 co-aggregation allowed the IP of RACK1–Lck complexes, which contain detectable levels of pY394^Lck^. Their highest levels were observed at 5–10 s after activation (Figure 5A, pY394^Lck^ panel, and Figure 5B), whereby the latter time point correlated with the peak of enrichment of Lck in these complexes (Figure 5A, Lck panel, and Figure 5C). This would suggest that a TCR/CD4-induced increase in pY394^Lck^ precedes and thus predicates the formation of RACK1–Lck complexes. However, the activation-induced RACK1–Lck complex formation was not ablated in T-cells that were pretreated with the SFK kinase inhibitor, PP2, which effectively inhibited the activation-induced global tyrosine phosphorylation of TCR downstream substrates (Figure 5D, 4G10 bottom panel, right lane, PP2+) and blocked the activation-induced enhancement of pY394^Lck^ levels (Figure 6D, pY394^Lck^ panel, PP2+). Together, these biochemical results strongly suggest that the physical interaction between Lck and RACK1 is independent of the activation status of Lck. However, under normal circumstances during TCR/CD4 engagement, upon which the preactivated pool of Lck is significantly increased (56), these complexes contain a sizeable fraction of pY394^Lck^ (Figure 5A).

### Identification of Additional Components of RACK1 Complexes in CD4^+^ T-Cells

Our observation that the formation of RACK1–Lck complexes was dependent on both the SH2 and SH3 domain of Lck (Figure 4E) suggested that this interaction could be indirect and involved additional structural components. Consistent with this notion are results from our pull-down experiments using *in vitro* translation that failed to show direct RACK1–Lck binding (data not shown). To gain added insight into the complexity of RACK1–Lck interaction, we first assessed the presence of additional components in RACK1 immunoprecipitates from activated primary CD4^+^ T-cells. To recognize their presence in immunoprecipitated RACK1 complexes, we took advantage of the fact that TCR signaling is a tyrosine phosphorylation (pY)-driven event, and thus, at least those components of RACK1 complex which change their pY status could be readily detectable.

As illustrated in Figure 6A, several tyrosine phosphorylated proteins co-immunoprecipitated with RACK1. Specifically, we found four major phosphoproteins in activated T-cells, three of which possessed the molecular weights (MWs) of ~100, ~70, and ~38 kDa, and their pY status was associated with RACK1 in an activation-inducible manner. The fourth phosphoprotein which possessed an MW of ~56 kDa matched the MW of Lck. To reveal the identity of the former three phosphoproteins, the corresponding areas from a coomassie-stained gel were excised and their protein content subjected to MS analysis (Table S1 in the Supplementary Material). Due to the fact that RACK1 is a scaffold protein that plays an essential role in transcription, epigenetics, and translation as well as binds diverse signaling and structural proteins, we zoomed in on those proteins that potentially act outside these activities. Among them, we focused on proteins that were known to be involved in proximal TCR signaling and cytoskeleton regulation of the forming IS (Figure 6B). The capacity of these candidate proteins to bind RACK1 before and after T-cell activation was assessed. GADS, LASP1, and α-actinin-1 exhibited sizeable changes in the kinetics of interaction with RACK1 upon TCR/CD4 co-aggregation. Notably, while α-actinin-1 binds to RACK1 with increasing intensity (from 0 to 90 s, Figure 6C, upper panel), the level of RACK1 interaction with GADS and LASP1 was diminished with similar kinetics over the time tested (Figure 6C, GADS and LASP1 panels). RACK1–GADS complex formation in resting primary T-cells was microscopically corroborated by their colocalization in resting T-cells (Figure S3 in Data Sheet 1 in the Supplementary Material).

### Only Kinase Active Lck–RACK1 Complexes Bind α-Actinin-1

While RACK1 interactome involves unusually high number of partners (BioGRID database; http://thebiogrid.org), the binding of α-actinin-1 to RACK1 complexes is an original finding. Actinins are primarily considered to be actin-cross-linking proteins but can also link transmembrane proteins to the cytoskeleton and membrane trafficking events (57). Our data showed that in resting CD4^+^ T-cells, α-actinin-1 inducibly associates with RACK1 upon TCR/CD4 co-aggregation (Figure 6D, TCRβ/CD4, 0 versus 10 s, α-actinin-1 panel, and Figure 6E). However, as described above, while RACK1–Lck complex formation proceeds even in the presence of SFK inhibitor PP2 when Lck kinase activity is severely compromised (Figure 6D, TCRβ/CD4, 10 s/PP2+, pY394^Lck^ panel), binding of this complex to α-actinin-1 is blocked (Figure 6D, last lane, α-actinin-1 panel, and Figure 6E). This advocates for a scenario in which TCR/CD4 co-ligation induces the formation of complexes which contain RACK1 and kinase active Lck (Figure 5A), the latter required for linking these complexes to α-actinin-1 component within the cytoskeleton. If Lck activity is blocked, the formation of RACK1–Lck complexes still proceeds, but α-actinin-1 is not engaged.

### Destabilization of Microtubules Prevents RACK1–Lck Complex Formation

**Figure 7:**
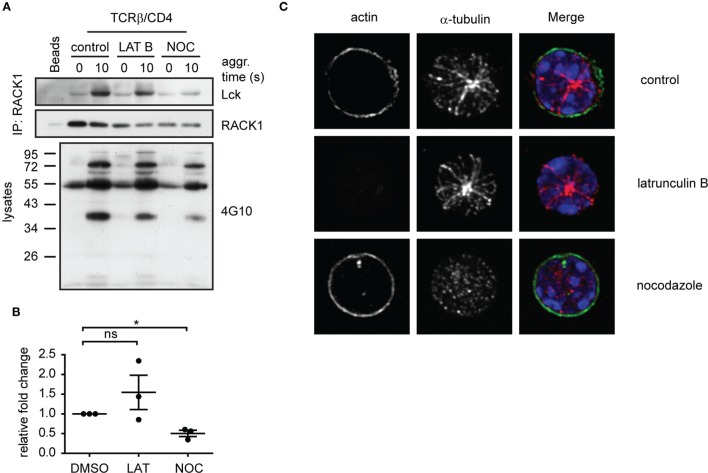
**Functional microtubular network is important for RACK1–Lck complex formation upon T-cell activation**. **(A)** CD4^+^ T-cells were pretreated with latrunculin B (LAT B), nocodazole (NOC), or DMSO (control) and then activated for the indicated period of time followed by lysis in TNE lysis buffer. RACK1 was immunoprecipitated and blotted against Lck and RACK1. Samples from lysates before IP were boiled and subjected to pY western blot analysis with 4G10 antibody. Blots are representative of three independent experiments. **(B)** Statistical analysis of **(A)**, Lck panel, represents the relative fold-change of RACK1 co-immunoprecipitated Lck normalized to total RACK1. The untreated control samples were given reference value “1.” The statistical analysis presented as mean ± SD was performed using the Student’s two-tailed *t*-test, *p < 0.05, n.s., not significant. **(C)** CD4^+^ T-cells treated with DMSO (control); nocodazole or latrunculin B were pre-warmed at 37°C and fixed by PFA on polylysine coverslips. Tubulin (red) and actin (green) were visualized by confocal microscopy. Nuclei were stained with DAPI (blue). Representative images show the maximal intensity projection (MIP) of *z*-stacks.

The timely and spatially coordinated complex formation between Lck and RACK1 in heavy DRM, their co-redistribution to the forming IS and linkage to α-actinin-1 suggest that the cytoskeletal network is actively involved. We have also reported that the activation-induced translocation of Lck to light DRMs is blocked by nocodazole, an inhibitor of microtubular assembly (14). To further investigate the potential involvement of RACK1 in the microtubular network-assisted translocation of Lck, we assessed the effect of nocodazole treatment on RACK1–Lck complex formation. The results showed that nocodazole, but not latrunculin-mediated inhibition of the actin cytoskeleton, effectively blocked the formation of RACK1–Lck complexes (Figure 7A, Lck panel, and Figure 7B). Of note, while lantrunculin and nocodazole treatment effectively disrupted the actin and microtubular network, respectively (Figure 7C), the activation-induced global tyrosine phosphorylation in the presence of these inhibitors was comparable to the untreated control sample (Figure 7A, bottom panel, 4G10). These data potentially provide a mechanistic explanation for the activation-dependent redistribution of Lck to light DRMs by virtue of linking the TCR/CD4-Lck complex to the microtubular cytoskeletal network *via* RACK1. This also suggests that at the very early stages of T-cell engagement, actin cytoskeleton does not affect Lck mobility even though α-actinin, which can potentially crosslink actin microfilaments, is already part of the Lck redistribution machinery.

### Knockdown of RACK1 Hinders Activation-Induced Lck Translocation to LR

**Figure 8:**
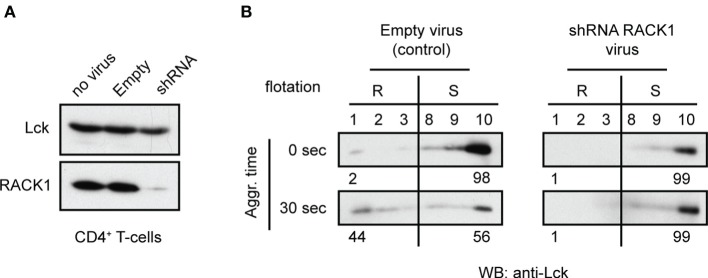
**Adenovirus mediated knockdown of RACK1 impedes translocation of Lck to light DRMs**. CD4^+^ T-cells from TgCAR transgenic mice were infected with either empty control virus (Empty) or with a mixture of adenoviral RACK1-targeting shRNA constructs (shRNA) or were not infected (no virus). **(A)** The effectiveness of RACK1 downregulation is shown 96 h after infection. Cells were harvested and probed with anti-Lck and anti-RACK1. **(B)** CD4^+^ T-cells infected with Empty virus (control) or shRNAs RACK1 virus were activated by TCR/CD4 co-aggregation (30 s), or not (0 s), and the redistribution of Lck to light DRMs was assessed. Raft (#1–3, R) and soluble (#8–10, S) fractions were probed with anti-Lck. Numbers represent relative distribution of Lck to these fractions. Blots are representative of two independent experiments.

We next attempted to evaluate the potential functional relevance of RACK1–Lck interaction for the redistribution of Lck to light DRMs (16). CD4^+^ T-cells from TgCAR transgenic mice expressing the receptor for adenovirus on T-cells were infected with adenovirus containing shRNAs to downregulate RACK1 (Figure 8A). After 96 h of adenovirus infection, the cells were harvested and activated by TCR/CD4 co-aggregation. The distribution of Lck to light DRMs was then compared to cells that were infected with a mock construct. Regardless of the technical caveats that are associated with our limited ability to consistently generate viable RACK1 knockdown (KD) immune cells (see commentary in Figure S4 in Data Sheet 1 in the Supplementary Material), our data showed that in activated T-cells, the reduced levels of RACK1 correlated with diminished redistribution rate of Lck to light DRMs (Figure 8B). These results points to the potential involvement of RACK1-based multiprotein signaling network in Lck redistribution during proximal T-cell signaling.

## Discussion

The overarching goal of our investigation was to provide the initial insight into the molecular mechanism involved in the early recruitment of Lck to light DRMs and the forming IS. As targeting Lck to light DRMs predicates enhanced TCR-mediated IL-2 production (21) and alternations in the association of Lck with accessory molecules within light DRMs supports abnormal T-cell signaling in autoimmune diseases (58), elucidation of the nature of this process on the molecular level represents a topic of academic and clinical interest. Our data provide compelling evidence that RACK1 orchestrates spatial redistribution of Lck *via* tethering to cytoskeletal elements.

Results presented in this study are the first to reveal and characterize the role of RACK1 in early T-cell activation. RACK1–Lck complex formation in primary CD4^+^ lymph node T-cells is activation-inducible, transient, and wanes shortly after activation. We have previously shown that only the pool of Lck that is associated with the HMW fractions translocates to light DRMs (14). Co-purification of RACK1 with these fractions in resting T-cells and the confinement of its activation-induced interactions with Lck to these fractions is consistent with its involvement in the shuffling of Lck to light DRMs. In addition, Lck and RACK1 co-redistribute with the same kinetics to the forming ISs. This argues for the existence of an underlying mechanism by which Lck and RACK1 are physically coupled and mechanistically transferred to these structures, likely *via* an association with common cytoskeletal elements (34, 59). In this context, our biochemical data showed that the formation of RACK1–Lck complexes, as well as the subsequent translocation of Lck to light DRM (14) depends on an intact microtubular network. While the relevant mechanism is obscure, there is precedence for the involvement of microtubular network in the proximal T cell signaling. As demonstrated previously, microtubules are located in close proximity to the cell membrane at the activation site and together with dynein regulate early microcluster transport and TCR signaling events (60). Thus, it is quite possible that microtubules can assist the binding of RACK1 and Lck, directly or indirectly, and *via* an unknown mechanism regulate their TCR/CD4-induced redistribution. Importantly, KD of RACK1 in primary CD4^+^ T-cells profoundly hampered the translocation of Lck to light DRMs. Thus, our data demonstrate that RACK1 fulfills the role of an adaptor protein that is involved in the regulation of Lck redistribution to light DRMs through the linking of TCR/CD4–Lck to the cytoskeletal network.

It has been previously reported that a Src–RACK1 interaction occurs through the binding of Src–SH2 domain to the tyrosine in position 246 (Y246) in the sixth WD40 domain of RACK1, which is phosphorylated by Src itself (32). As Src and Lck share the same structural components and domain organization, one could assume that RACK1–Lck interaction should also be dependent on the SH2 domain of Lck. However, we were unable to detect tyrosine-phosphorylated residues (pY) on RACK1 10 s after TCR/CD4 co-aggregation (data not shown). Thus, it is unlikely that RACK1–Lck interaction is mediated *via* binding of Lck–SH2 to pY on RACK1. Unexpectedly, RACK1–Lck interaction was also abrogated in the SH3-inactivated mutant of Lck. This indicates an equal importance of these two domains in RACK1–Lck complex formation. However, the structural basis for RACK1 binding to SH3–Lck is uncertain. Alternatively, if these interactions are mediated through some intermediary, it would predict the formation of multiprotein complexes involving numerous protein–protein interactions. Our data support this scenario. Notably, in addition to RACK1, Lck redistribution machinery might include the adaptor protein GADS (61) and components of the cytoskeleton such as α-actinin (62) and LASP1 protein (63). Other potential components such as serine/threonine protein phosphatases PP1 and 2A, protein SEC13 homolog, F-actin capping protein, Annexin A2, and ERM proteins were detected (Table S1 in the Supplementary Material), but validation of their presence and potential function in RACK1 complexes in T-cell proximal signaling needs further experimentation.

Interestingly, our data showed that Lck formed complexes with RACK1, irrespective of its kinase activity status, suggesting that conformational changes of TCR and/or CD4 may play a role in their induction (64). While the underlying mechanism awaits some resolution, we also demonstrated that it is only when these complexes contain kinase active Lck, they recruit α-actinin-1. The binding of α-actinin-1 to RACK1–Lck complexes adds another layer of complexity to the schematic of TCR signaling. Notably, it has been previously reported that α-actinin-1 associates with the membrane fraction of mouse lymphocytes (65) and resides in heavy DRMs of in immune cells (66). It has been also shown that α-actinin binds directly to both phosphatidylinositol-(4,5) biphosphate (PtdInsP_2_) and PI-3 kinase which when activated, converts PIP_2_ to phosphatidylinositol-(3,4,5) triphosphate [PtdIns(3,4,5)-P_3_] at the inner leaflet of the plasma membrane (67). α-actinin-1 is also a target of activated tyrosine kinase (68), the nature of which, in T-cells, has not been elucidated. Interestingly, while α-actinin primarily acts to bundle actin filaments, this function upon the initiation of TCR signaling would be likely subjected to negative regulation by all the three mechanisms mentioned in this study. Notably, binding to α-actinin of PtdIns(3,4,5)-P_3_, which is generated by activated PI-3 kinase (69) and tyrosine phosphorylation with increased binding of Ca^2+^ to EF domains of α-actinin (70), would additively or synergistically reduce actinin’s affinity for actin. Thus, while TCR/CD4 engagement recruits α-actinin-1 to RACK1–Lck complexes, during proximal T-cell signaling, α-actinin-mediated actin bundling would be compromised. However, whether such a mechanism in T-cells is indeed operational is currently under investigation.

Our data from primary CD4^+^ T-cells also confirmed a previous finding that RACK1 physically associates with GADS in resting Jurkat T-cells (71). GADS and SLP76 are critical components of the signaling pathway which, upon TCR activation, inducibly bind to the phosphorylated scaffold protein LAT (61), which is responsible for subsequent actin cytoskeletal rearrangement (5). While speculative, a more plausible scenario would be that after TCR/CD4 triggering, tyrosine kinases Lck and ZAP70 are activated with the latter phosphorylating LAT at multiple sites (pY-LAT). The RACK1–GADS–SLP76 complex is then recruited to pY-LAT *via* SH2 of GADS, brought into the proximity of the TCR/CD4 complex and gains access to activated Lck. RACK1 then dissociates from GADS and forms multiprotein complexes which include several signaling components. Inclusion of kinase active Lck in this complex is predicated by the presence of an unperturbed microtubular cytoskeleton, which then mediates Lck redistribution to light DRMs, where Lck phosphorylates Fyn. This model is consistent with the kinetics of Lck translocation to light DRMs: TCR/CD4 co-aggregation-induced Lck enrichment in light DRMs reaches its maximum at 30 s, which correlates with the already decreasing amount of activation-induced RACK1–Lck complexes (16). Binding of α-actinin-1 to the RACK1 complex exclusively in the presence of kinase active Lck suggests that formation of RACK1–pY394^Lck^–α-actinin-1 module acts as a regulatory switch for the engagement of actin cytoskeleton upon productive TCR/CD4 triggering. A precise molecular mapping and structure–function analysis will be needed to dissect the parameters of this transient multiprotein complex formation and its interactome.

Our data also raise a fundamental question concerning the spatiotemporal regulation of interaction between microtubular and actin cytoskeleton during T-cell proximal signaling. We need to take into account evidence that not only the two filament systems interact with each other *in vivo* (72) but also that α-actinin specifically plays an integral role in the cooperative regulation of microtubular and actin cytoskeleton dynamics (73–75). In addition, as both RACK1 and α-actinin have been implicated in costimulatory and/or adhesion signaling which closely follows the TCR triggering event (71, 76, 77), it would be not entirely surprising that their TCR-induced complex formation would integrate signals from multiple receptors, including TCR/CD4, CD28, and integrins, and orchestrate the complex cooperative microtubular and F-actin cytoskeleton rearrangement. Importantly, as during early phases of TCR signaling, due to the adverse effect of tyrosine phosphorylation of α-actinin and increased levels of Ca^2+^ and PtdIns(3,4,5)-P_3_, actin cytoskeleton would not be able to effectively engage, allowing T-cell membrane relaxation, rapid redistribution of signaling membrane protein to the forming IS, and coalescence of various types of membrane LRs, including redistribution of Lck to light DRMs. Later, once the initial wave of secondary messengers wanes, actinin engages and bundles filamentous actin so that the formation and maturation of actin architecture surrounding IS can be accomplished.

Taken together, data presented in this study advocate for the existence of a novel mechanism that integrates the engagement of TCR/CD4 receptors with cytoskeletal network *via* forming RACK1-based multiprotein network. While there is no doubt that more experimentation is necessary to fully elucidate its composition, structure, dynamics, kinetics, and the type of activation-dependent behavior, to the best of our knowledge, these are the first data that revealed its involvement in proximal T cell signaling with potential impact on the activation-induced repartitioning of Lck.

## Ethics Statement

The study was approved by the Ethics Committee of the Institute of Molecular Genetics under the number #175/2011. The use of animals was also approved by the Academy of Sciences of the Czech Republic.

## Author Contributions

DF initiated and designed the experiments, and with OB analyzed the data. OB performed most of the experiments. JV and AB were involved in confocal microscopy and IP experiments. MD, OB, and JM prepared NIH3T3 transfectants, assisted with FACS analysis, and performed all animal work. PŘ and JS prepared samples and preformed MS TOF/TOF analysis and data mining. OB, JM, and DF wrote the initial draft of the manuscript, and DF finalized the manuscript.

## Conflict of Interest Statement

The authors declare that the research was conducted in the absence of any commercial or financial relationships that could be construed as a potential conflict of interest.

## Supplementary Material

Video S1 **Time-lapse imaging RACK1 redistribution into immunological synapse upon T-cell activation**. WT Jurkat T-cells expressing RACK1-EGFP (green) constructs were mixed with SEE pulsed RAJI B-cells and the formation of IS was observed by live cell imaging microscopy.

Video S2 **Time-lapse imaging of Lck and RACK1 co-redistribution into immunological synapse upon T-cell activation**. Lck-deficient JCAM1.6 Jurkat T-cells co-expressing Lck-CFP (red) and RACK1-mCitrine (green) constructs were mixed with SEE pulsed RAJI B-cells (blue) and the formation of IS was observed by live cell imaging microscopy.
