# Supplementary material for: TCR Triggering Induces the Formation of Lck–RACK1–Actinin-1 Multiprotein Network Affecting Lck Redistribution
Source: Front Immunol. 2016 Oct 27;7:449. doi: 10.3389/fimmu.2016.00449 (PMC5081367; doi:10.3389/fimmu.2016.00449)
Supplement: Supplementary file 1 [file data_sheet_1.pdf]

Supporting Information

TCR triggering induces the formation of Lck-RACK1-actinin-1 multiprotein network affecting Lck redistribution

Ondřej Ballek, Jan Valečka, Martina Dobešová, Adéla Broučková, Jasper Manning, Pavel Řehulka, Jiří Stulík and Dominik Filipp

Content in pdf format:

Supplementary Figure 1. Quantification of concentricity and apposition of RACK and Lck (or GADS) in primary naïve $CD4^+$ T-cells

Supplementary Figure 2. Preparation of NIH3T3 fibroblast cell lines expressing comparable levels of various Lck proteins

Supplementary Figure 3. Distribution of GADS and RACK1 in primary $CD4^+$ T-cell

Supplementary Figure 4. Comments on technical difficulties associated with RACK1 knockdown experiments in immune cells

Content in mov format:

Supplementary Video 1. Time-lapse imaging of RACK1 redistribution into immunological synapse upon T-cell activation.

Supplementary Video 2. Time-lapse imaging of Lck and RACK1 co-redistribution into immunological synapse upon T-cell activation.

Content in excel format:

Supplementary Table 1. Identification of additional components of RACK1-Lck complexes

Supplementary Figure 1.

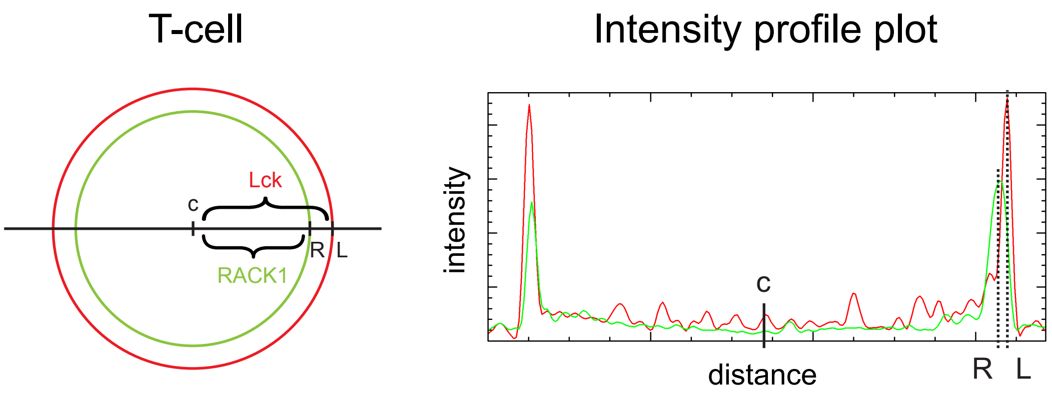

Quantification of concentricity and apposition of RACK1 and Lck (or GADS) in primary naive CD4$^+$ T-cells | To analyze concentricity and apposition of RACK1 and Lck [or GADS)], the distance from the centroid of the cell to the cell edge was measured using fluorescent intensity profile plot. Specifically, using ImageJ program a centroid of the cell [point c] was determined (left figure). A line was drawn through the centroid and the fluorescent intensity profile plot for both channels [e.g. red and green] was calculated (right panel). The distance from the centroid to the point of highest intensity value for each channel [e.g. RACK1 (R) and Lck (L)] was then measured. All microscopic quantitative analyses were performed by ImageJ program. Statistical analysis was performed with GraphPad Prism 5 using a paired two-tailed t-test (Fig 1d, Suppl. Figure 4).

Supplementary Figure 2.

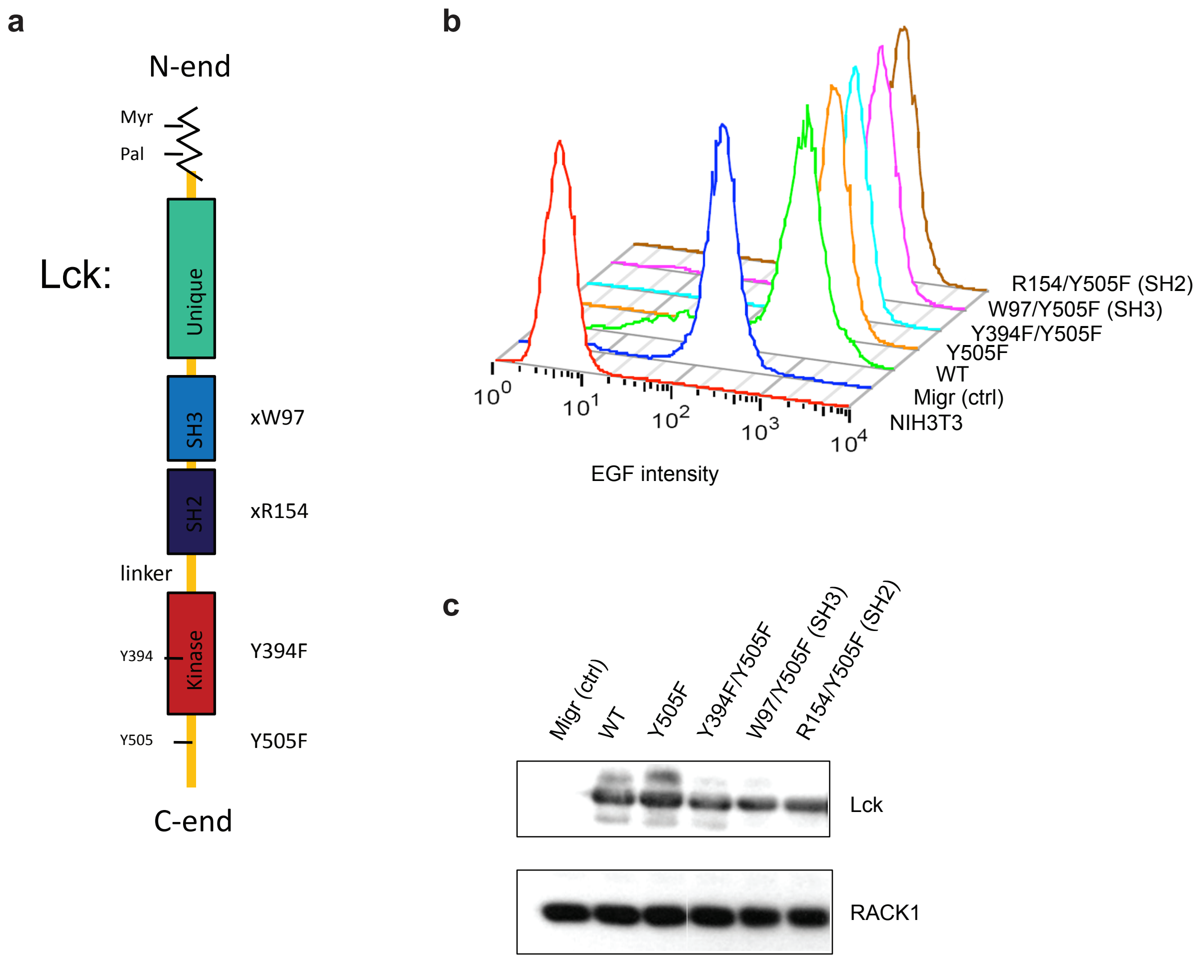

Preparation of NIH3T3 fibroblast cell lines expressing comparable levels of various Lck mutants | **(a)** Lck domain structure with indicated point mutations/deletion that affect the function of SH3, SH2 and catalytic domains and the regulatory C-terminal region (not used in this study) is depicted (the construction of these mutants was described elsewhere (Filipp et.al., 2008). **(b)** A histogram showing the comparable intensity of EGFP expression among Lck infectants measured by flow cytometry. **(c)** Western blot analysis performed on total cell lysates revealed comparable protein levels of Lck and RACK1 among indicated Lck infectants.

Supplementary Figure 3.

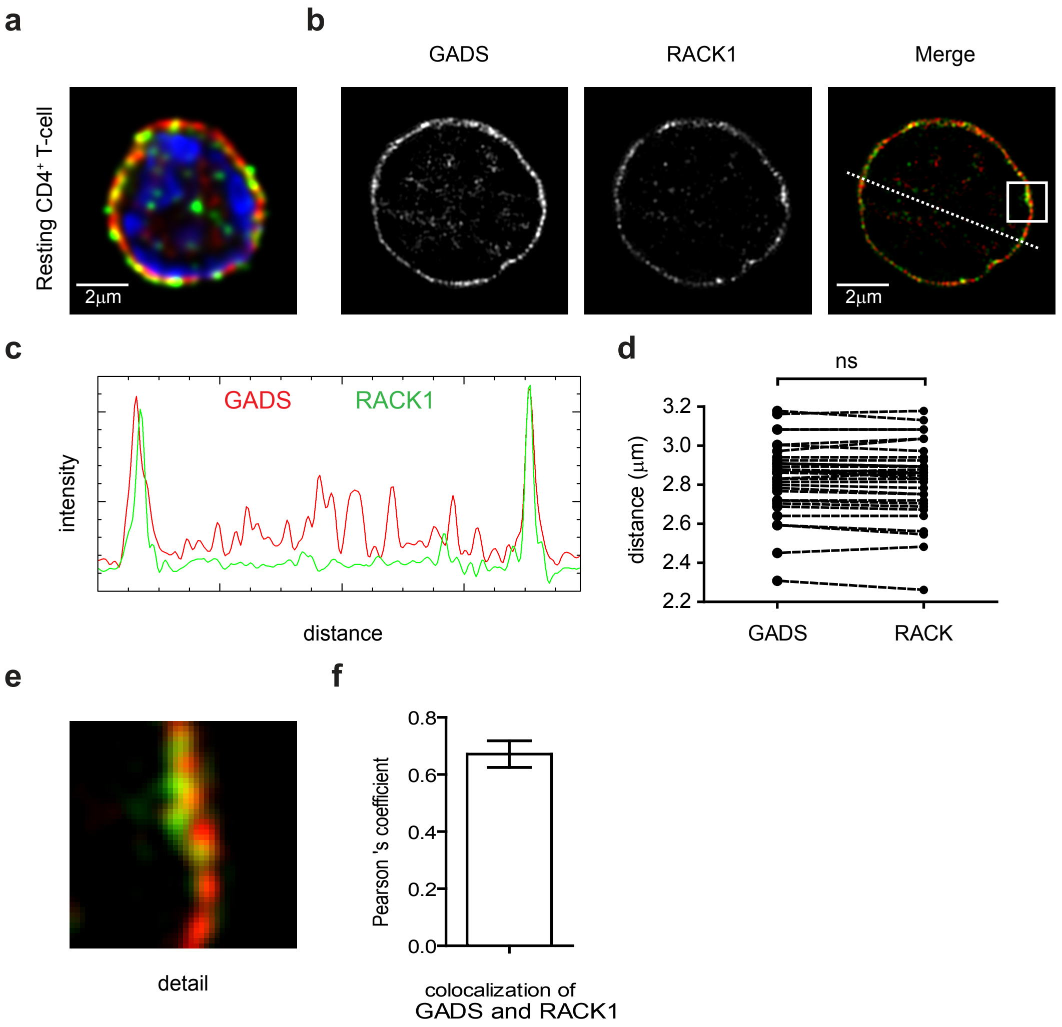

Suppl Figure 4 | Subcellular distribution of RACK1 and GADS in primary CD4⁺ T-cell. Fixed CD4+ T-cells were stained for RACK1 (green), GADS (red) and nuclei (blue) and visualized by confocal microscopy **(a)** or super-resolution N-SIM microscopy **(b)**. **(c)** Fluorescence intensity profile plot of RACK1 (green) and GADS (red) along the dotted line shown in the merged image of figure b. **(d)** statistical analysis of juxtaposition of RACK1 and GADS which shows a comparable distance of RACK1 and GADS from the cell centroid to its periphery; (n=30). **(e)** Magnification of the rectangle insert from the merge image presented in (b) showing a considerable overlap of RACK1 and GADS. **(f)** The bar graph represents the statistical analysis of RACK1 and GADS colocalization using Pearson's colocalization coefficient; (n=20 cells). Error bars denote standard deviation.

Supplementary Figure 4.
# - commentary

Comments on technical difficulties associated with RACK1 knockdown experiments in immune cells | In regards of the functional importance of RACK1 signaling network, the RACK1 knockdown (KD) experiments are considered to be critical. However, several technical problems and caveats associated with such approach precluded us to fully explore such opportunity. First of all, the absence of RACK1 or its diminished levels are detrimental for any type of cell (RACK1 is embryonically lethal). This is most likely due to its indispensible role in ribosomal assembly and regulation of translation (on top of other functions in intracellular transport). The KD experiment, highlighted in Figure 8, was performed at least eight times. Primary naïve CD4+ T-cells contain relatively high amounts of RACK1 protein (Fig. 1 and 2) and due to their slow metabolism, it takes approximately 3-4 days to reduce its amount by adenovirus-mediated KD approach to suitable low levels. However, when the amount reaches such a low threshold, cells rapidly induce and undergo apoptosis. It is extremely difficult to time this process in the way that RACK1-deprived cells are still able to respond to TCR/CD4 stimulation. After the lengthy 3-day infection procedure we sorted out live cells and monitored their signaling capacity via 4G10 phosphotyrosine specific western blotting (**Supplementary Figure 4, panel 1**). Out of these 8 experiments, only two batches of RACK KD CD4+ T-cells were signaling competent (**Fig. 8 and Supplementary Figure 4, panel 2 and 3, which showed the results of these two independent experiments**), the remaining six were compromised. We also attempted to perform the activation assay with signaling competent RACK1 KD T-cells using plate-immobilized anti-CD3 and soluble anti-CD28 antibodies, with the aim to measure the activation-induced expression of CD69 (after 4 hours) or CD25 (after 24 hours). However, due to a dramatically high degree of apoptosis, the results were inconclusive. As an alternative approach, we designed an experiment to generate T-cells from RACK1 KO ES cells which are available at the Comp repository at UC Davis, USA (www.komp.org) by co-culturing them with OP-9 delta-like1 stromal cells (Schmitt TM, Zúñiga-Pflücker JC. Immunity, 2002 Dec; 17(6):749-56. However, all ES clones that the KOMP depository make commercially available, failed viability, growth, and morphology testing. In addition, obtaining live video images from Jurkat model system (Fig. 3), we attempted to prepared RACK1 KD Jurkat T-cell and assess the mechanics of Lck in RACK1 absence. However, and as explained above, this is a very precarious exercise due to the drastically lowered viability of cells with reduced levels of RACK1. We have attempted to use siRNA to silence RACK1 expression in Jurkat T-cells. We used various siRNA sequences and transfection protocols. Unfortunately, these cells have very little tolerance to changes in RACK1 levels and die before RACK1 reaches <20-30% of its physiological levels, precluding us from performing this type of experiments. We observed similar results using the shRNA approach. This is very frustrating and unfortunate as Jurkat-cells could serve as an excellent model for studying RACK1-dependent Lck redistribution mechanism. In conclusion, at this time, there are only very limited resources and tools to genetically manipulate the expression of RACK1 gene. New tools will be necessary to further develop the research with functionally promiscuous proteins like RACK1, especially, if such functions relate to critical role in the regulation of translation. Needless to say that even the conditional cre-driven T-cell-specific RACK1 KO would not help to resolve this conundrum.

Supplementary Figure 4.

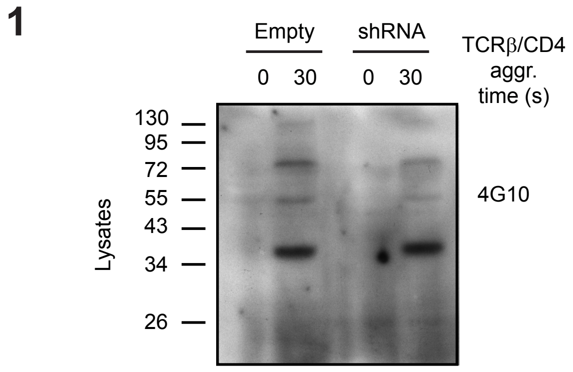

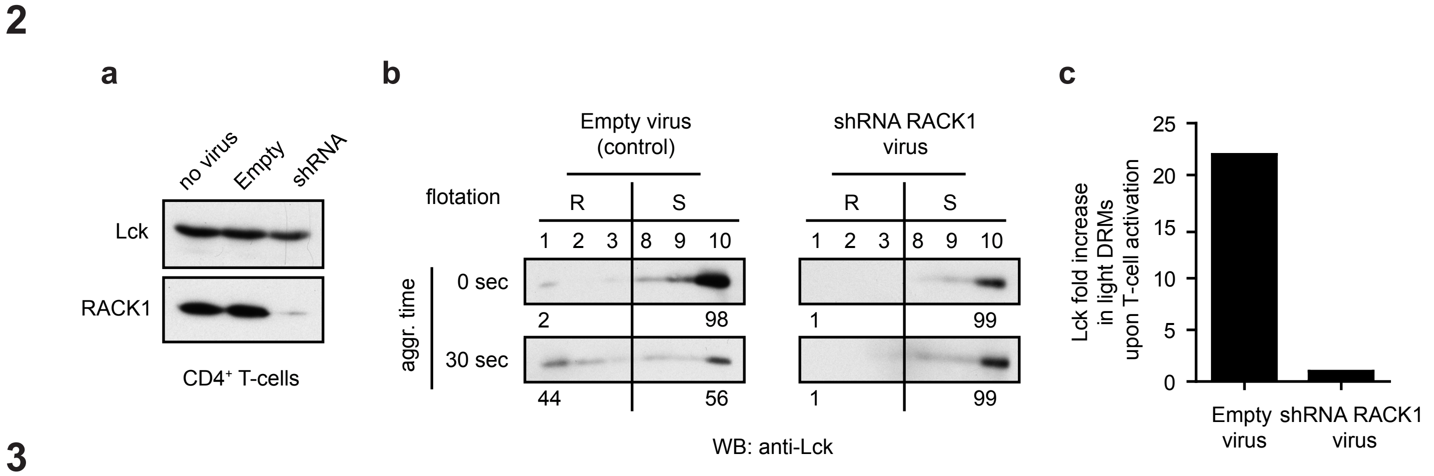

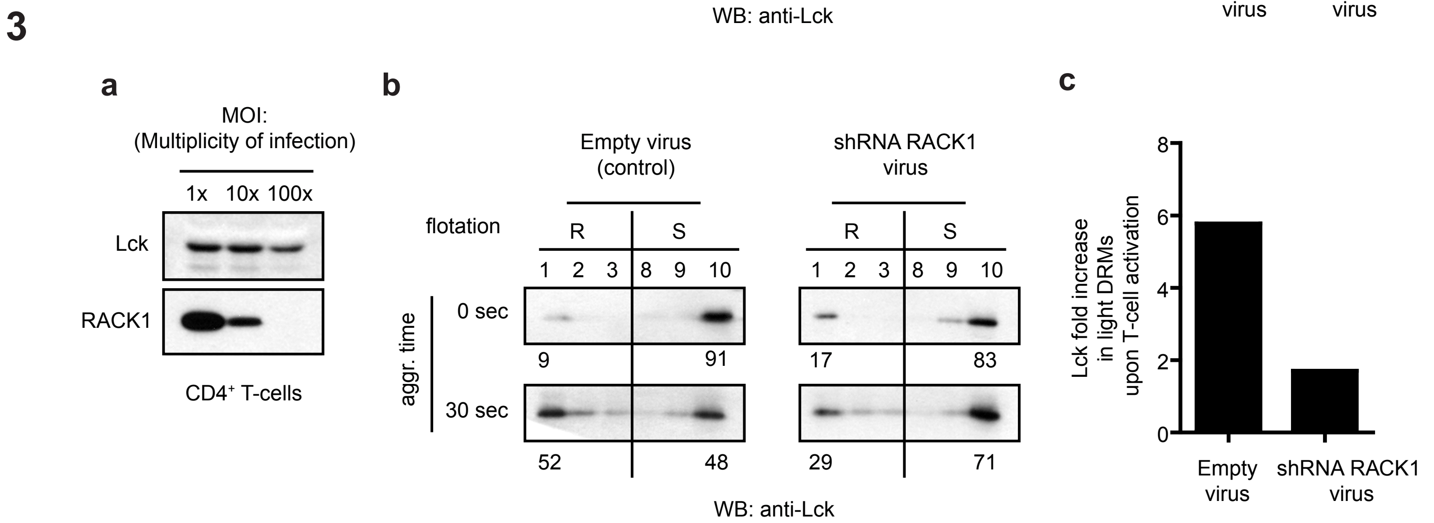

Supplementary Video 1

Time-lapse imaging RACK1 redistribution into immunological synapse upon T-cell activation.

WT Jurkat T-cells expressing RACK1-EGFP (green) constructs were mixed with SEE pulsed RAJI B-cells and the formation of IS was observed by live cell imaging microscopy.

Supplementary Video 2

Time-lapse imaging of Lck and RACK1 co-redistribution into immunological synapse upon T-cell activation.

Lck-deficient JCAM1.6 Jurkat T-cells co-expressing Lck-CFP (red) and RACK1-mCitrine (green) constructs were mixed with SEE pulsed RAJI B-cells (blue) and the formation of IS was observed by live cell imaging microscopy.
